# Long-term waist circumference trajectories and body mass index with all-cause mortality in older Chinese adults: a prospective nationwide cohort study

**DOI:** 10.1186/s13690-022-00861-y

**Published:** 2022-09-10

**Authors:** Ruru Liu, Shaonong Dang, Yaling Zhao, Hong Yan, Yuewen Han, Baibing Mi

**Affiliations:** 1 Department of Disinfection, Xi’an Center for Disease Control and Prevention, Xi’an, Shaanxi Province 710054 China; 2 Department of Epidemiology and Biostatistics, School of Public Health, Xi’an Jiaotong University Health Science Center, No.76, Yanta West Road, Xi’an, Shaanxi Province 710061 China

**Keywords:** WC, Mortality, Longitude trajectory, Older Chinese, Cohort study

## Abstract

**Backgrounds:**

Abdominal obesity has been linked to risk of mortality, but whether and how trajectory of waist circumstance (WC) underpins this association remains unclear. The study aimed to identify long-term WC change trajectories and examine their association and joint effect with body mass index (BMI) on mortality among Chinese older adults.

**Methods:**

This present study included participants 60 years of age or older from China Health and Nutrition Survey (CHNS) from 1991 to 2015. The duration of follow-up was defined as period from the first to latest visit date attended with information on mortality, end of follow-up, or loss to follow-up (censoring). Latent class trajectory analysis (LCTA) was used to assess the changes of WC trajectories overtime. Cox proportional hazard models were used to assess hazard ratios (HRs) and corresponding 95% confidence internal (CIs) for mortality.

**Results:**

A total of 2601 participants with 8700 visits were included, and 562 mortality (21.6%) occurred during a median follow-up of 8.7 years. Using a group-based modeling approach, four distinct trajectories of WC change among Chinese older adults were identified as loss (13.5%), stable (46.8%), moderate gain (31.2%) and substantial gain (8.5%). With WC stable group as reference, the multivariable adjusted HRs for mortality were 1.34(95%CI:1.01-1.78) in loss group, 1.13(0.91-1.41) in moderate gain and 1.54(1.12-2.12) in substantial gain group. Compared with participants with normal BMI at baseline and maintained WC stable, the risk of mortality generally increased for all WC change group in initial overweight/obesity individuals, and the highest risk were observed for WC loss and stable pattern (HR:2.43, 95%CI: 1.41–4.19; HR:1.67 (1.07–2.60)).

**Conclusions:**

In older Chinese, both long-term WC loss and substantial gain conferred excess risk for mortality. The baseline BMI might modify the effect as overweight individuals had a greater risk imposed by WC loss than those in normal weight. Maintaining stable WC and normal weight might be necessary to reduce the risk of mortality.

**Supplementary Information:**

The online version contains supplementary material available at 10.1186/s13690-022-00861-y.

## Background

The prevalence of obesity and overweight is high and increasing dramatically in all age groups [1, 2]. In China, it was estimated that 85 million adults (95% CI: 70 million-100 million]) aged 18–69 years were in obesity measured by BMI in 2018, which had tripled from 2004 [3]. Meanwhile, visceral obesity measured by waist circumference (WC) is common among Chinese population, even in those with normal BMI (30.3% in 2011) [4]. Notably, aging was generally associated with higher WC and redistribution of body fat to the abdominal region were often reported in the older [5]. Therefore, it is important to evaluate the long-term trend and distribution of WC among the older adults, considering the huge ageing population.

The association between WC and all-cause mortality has been a topic of debate. It is well-known that potential causal association between higher WC and all-cause and cardiovascular mortality was supported by prospective observational studies [6]. And even among those in normal BMI range, WC was positively associated with mortality in older adults [7, 8]. Furthermore, evidence was increasingly accumulating that WC change might be associated with additional health outcomes, compared to static weight status [7, 9]. Most of them implied that the WC change trajectories might vary across race and age, and suggested that higher mortality might attribute to steeper increase in the slope of WC [10–12].These efforts were valuable but far from conclusive because of methodological limitations, as most previous studies defined WC change as the difference in WC measures between two time points, or assumed the WC-mortality link over time was simple linear. Moreover, they tended to interpret insufficiently for the possible interaction with initial BMI (the most commonly used measure of weight status), although previous cohort studies highlighted the importance of combing both WC and BMI for risk assessment with mortality [13, 14].Yet, data is still limited on the long-term WC-mortality link among older Chinese adults, who are combating with severe epidemic of abdominal obesity [15].The two cohort studies reported a U-shaped association between WC change and mortality in older Chinese [12, 16]. However, both of them calculated the WC change as the proportion disparity between current and initial WC, which could not identify the true change circle. In present study, we aimed to extract WC change trajectories over time among older Chinese based on a nationwide population-based cohort study. We tried to examine association between WC change patterns and mortality, and outline the specific WC change trajectory related to higher risk for guiding adiposity and associated health risk. We also attempted to assess the potential joint effect of WC change and BMI at baseline with morality.

## Materials and methods

### Study population

Data used in current study was from China Health and Nutrition Survey (CHNS), which was an ongoing open-cohort, collaborative study to examine the effect of social and economic transformation of Chinese society on nutrition and health status in this population. The open database, study materials and acknowledgement is available at the website (www.cpc.unc.edu/projects/china). A multistage random cluster process was used to draw a sample of about 7,200 households with over 30,000 individuals in 15 provinces. The first round of CHNS was conducted in 1989 and was subsequently conducted in 1991, 1993, 1997, 2000, 2004, 2006, 2009, 2011 and 2015. A detailed description of study design and procedures has been reported elsewhere [17]. All participants signed the informed consent and this study was approved by the institutional review committees of the National Institute of Nutrition and Food Safety, Chinese Center for Disease Control and Prevention, the University of North Carolina at Chapel Hill, and the China–Japan Friendship Hospital, Ministry of Health.

In present study, all work followed the Strengthening the Reporting of Observational studies in Epidemiology (STROBE) statement of cohort studies. The first survey wave was set as 1993 when complete WC information was collected. In brief, the data used were based on 8 waves of CHNS performed from 1993 to 2015. The flow diagram of participants was summarized in Supplemental Fig. 1. A total of 31,770 participants with 92, 497 visits were extracted from the original study. We excluded participants who were less than 60y or less than 2 visits in the follow-up period, pregnant women, and who were with outlying data (e.g., weight > 300 kg or < 20 kg). Finally, 2601 participants with 8,700 visits were available for the final analysis.

**Fig. 1:**
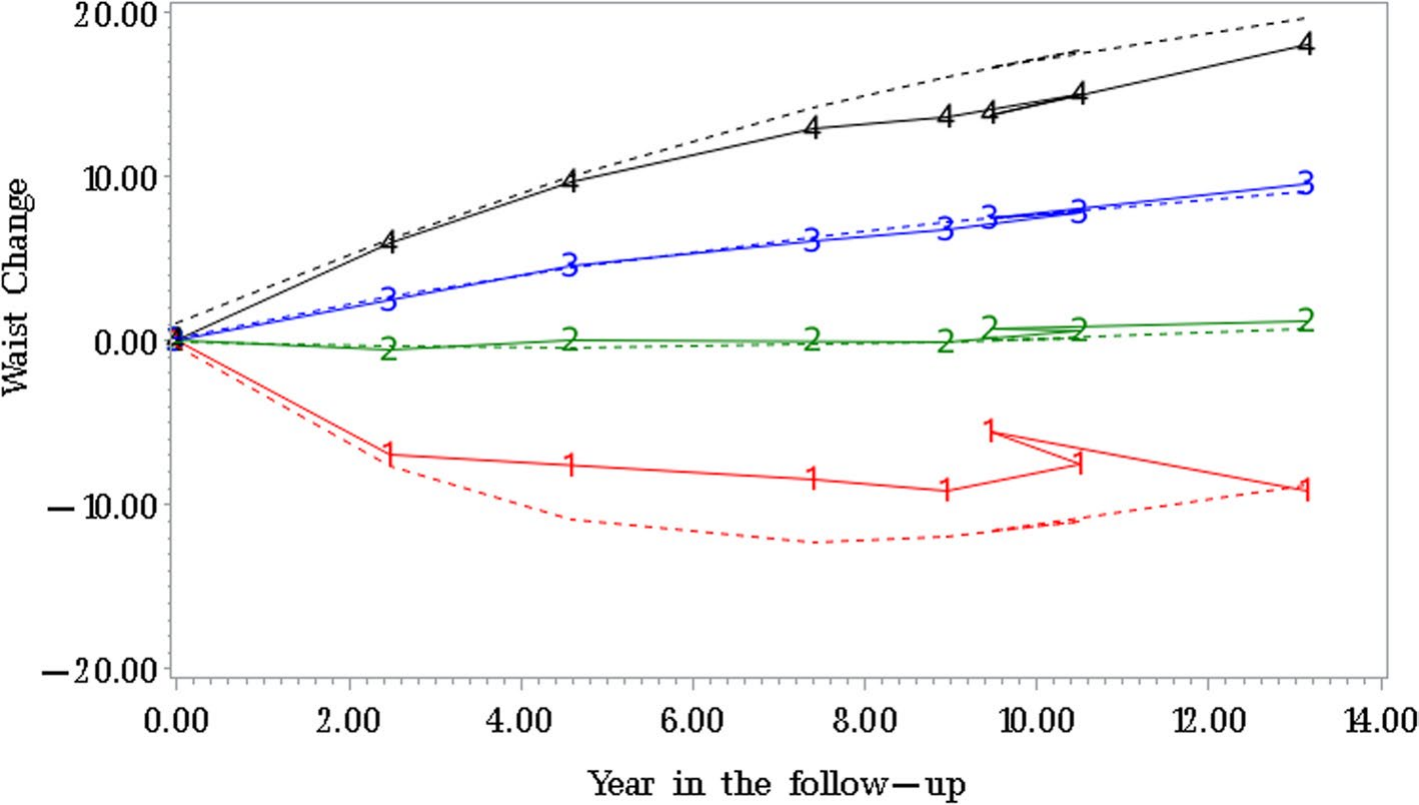
Trajectory modeling identified four distinct WC change patterns. For ease of interpretation, WC substantial gain trajectory are presented in black, moderate gain in blue, stable trajectory in green and WC loss trajectory in red. WC indicates waist circumference

### Definition of follow-up in the study

Individuals included in current analysis were followed prospectively from their first visit in the CHNS. The study was an open cohort, as participants might enter and leave in any wave, and those lost to follow-up in one wave were still likely to enter the survey in other waves. Therefore, the duration of follow-up duration was defined as period from the first to latest visit attended with information on mortality, end of follow-up, or loss to follow-up (censoring).

### Ascertainment of deaths

All-cause mortality was ascertained based on the report of household members in each survey year [18]. If a death case was duplicated in more than one survey wave, then the first report was chosen [18].

### Anthropometry measurement

Weight, height, and WC were measured by trained health care workers following standardized protocols as recommended by the WHO at each wave [17]. Weight was measured to the nearest 0.1 kg using a calibrated beam scale with participants wearing lightweight clothing, and height was measured to the nearest 0.1 cm using a portable stadiometer without shoes. WC was measured at a point midway between the lowest edge of the rib cage and the highest edge of the iliac crest in a horizontal plane using nonelastic tape [19]. Three measurements were taken for all indicators, and the averages were used for the analyses [19]. BMI was categorized as follows: lean, BMI < 18.5 kg/m^2^; normal weight, BMI was from 18.5 to 23.9 kg/m^2^; overweight, BMI was from 24.0 to 27.9 kg/m^2^; obesity, BMI ≥ 28 kg/m^2^ [20]. WC was classified as: WC < 80.0, 80.0 to 87.9, 88.0 to 93.9, ≥ 94 cm, corresponging to low, noraml, morderate-high and high WC for men; the cut point for women was < 72.0, 72.0–79.9, 80.0–87.9, ≥ 88, respectively [21]. And the last two was defined as central obesity.

### Trajectory of change in WC

We used latent class trajectory analysis (LCTA) implemented by SAS Proc Traj procedure, a group based modeling approach to identify subgroup sharing similar underlying trajectory of change [22, 23].We modeled WC change between each wave as current WC minus baseline WC overtime and repeated trajectory analysis with changing group number from 2 to 5, and performed both linear, quadratic and cubic of models. To determine the optimal model following standard guidelines [22], we considered several factors including the Bayesian Information Criterion (BIC), significance of polynomial terms, value of average posterior probability (entropy) and of group membership probability, leaning towards parsimony in number of trajectory groups [24]. For each trajectory, we aimed for groups with membership probabilities ≥ 5% [24]. Within each trajectory, the value of mean posterior probability of membership was ascertained, as it was at least 75.0% indicated enough internal reliability [25].Further information on parameter estimation of fitting WC trajectory model were available in Supplemental material. After WC change trajectories were determined, each individual was assigned exclusively to the trajectory group with the highest posterior probability. Trajectory membership was used as an indicator variable in present analyses.

### Covariates assessments

Evidence from large-scale cohort studies suggested that mortality risk and weight change might be confounded by demographics variables, lifestyle factor and physical measurement (BMI and blood pressure) [19, 26, 27]. According to these studies and a priori knowledge about CHNS data [28], a set of covariates were ascertained including socio-economic status (gender, age, enrollment year, education and income level, residence), lifestyle variables (smoking, drinking and physical activity), dietary energy intake (total energy intake) and baseline anthropometry (blood pressure, BMI and WC) and chronic diseases.

Information on socio-economic status and lifestyle factors were collected through standardized interview questionnaire. Income was measured in RMB and derived from total household. Smoker were assigned if they answer “yes” to the question “Have you ever smoked cigarettes (hand-rolled or device-rolled)”. Similarly, drinkers were defined if they consumed alcohol including beer, wine or other alcoholic beverage during the past year. Physical activity level was measured by a semi-quantitative question, including items of occupational, domestic, travel, and leisure time in this study. The intensity score, indicated by metabolic equivalent (MET) score, was calculated by multiplying frequency and duration of the activity converted to per day [29]. Dietary energy intake was estimated via 3 consecutive 24-h recalls. The individual and household were asked to record each type and weight of food they consumed during 3 consecutive day [30]. Chronic diseases included diabetes, myocardial infarction, stroke and cancer, and they were diagnosed by trained clinicians and self-reported by participants in each survey wave.

### Statistical analysis

We calculated the follow-up person years from the date of returning the individuals first enrolled to the date of mortality or the end of cohort, whichever came first. Baseline demographic and health-related variable were summarized across different WC change trajectories, with number and percentage for categorical variables and mean and S.D. for continuous ones. Chi-square test and ANOVA were used to compare baseline characteristic differences. Cox proportional hazard regression was used to assess the WC-mortality link, with WC stable trajectory as reference. We elevated the proportional hazards assumption by creating interaction term of follow-up time and main variables including WC change trajectories. Results of likelihood-ratio test indicated no significant, consistent with visual inspection of log–log plots. Hazard ratios (HRs) and corresponding 95% confidence intervals (CIs) were adjusted for covariates including age (continuous), urbanization (urban, rural), gender, education (never, < 6 years, 6–8 years, 9–11 years, >  11 years) and income level (continuous), enrollment year (continuous), initial BMI (lean, normal, overweight and obesity) and WC (continuous), initial SBP/DBP (continuous), smoking status (no, yes), drinking status (no, yes), physical activity (categorized by median value, 4.3 MET*hours/day) and dietary energy intake (categorized by median value, 1964.5 kcal/d). Consistent with previous studies [28], we completed the main analysis by assuming that variables were missing completely at random (MCAR). We examine the interaction of WC change trajectories and BMI at baseline by creating a cross product of them. In the regression model, this variable was significantly associated with event (Wald χ^2^ = 7.910, *P* = 0.005). Likelihood ratio test comparing with and without this variable conforms this result with significance. Thus, we stratify the sample by baseline BMI status to better understand this relationship. We repeated Cox proportional hazard regression, with WC stable trajectory and normal weight at baseline as reference. Furthermore, we conducted subgroup analysis to explore the possible heterogeneity of association, according to the baseline characteristics as gender, BMI status, urbanization, smoking status, drinking status, physical activity and dietary energy intake. For each of these variables, we tested for potential effect modification using likelihood ratio tests. Due to the potential for type I error caused by multiple comparisons, the interpretation of subgroup analyses should be treated as exploratory [31].

We also conducted several sensitivity analyses to evaluate the robustness of the findings. First, we excluded participants with 3 times or less to test applicability of favorable models and its association with mortality. Second, we used subsample of individuals without a history of chronic diseases (diabetes, myocardial infarction, stroke or cancer), to accommodate potential residual confounding from pre-existing disease. Third, we completed multiple imputation using the chained equations models. The predictors in multivariate models included WC change trajectories, hypertension and other demographics, anthropometry and health behavior variables. We used linear regression models for continuous variables and discriminant functions for categorical variables to impute values. A total of 20 datasets were generated, as the total missing across these variables was lower than 15%. We repeated the analysis in each augmented dataset, and calculated parameter estimates the 20 sets and their SEs by the Rubin method. Finally, to assess the effect of baseline WC on the association between WC change trajectories and mortality, we estimated HRs of mortality according to baseline WC (non-central obesity and central obesity), with WC stable trajectory and normal WC at baseline as reference. All analyses were conducted with SAS 9.4 (SAS Institute, Cary, NC). Two-sided tests were used and *P* < 0.05 was considered statistically significant.

## Results

### Baseline characteristics of the study population

A total of 2601 individuals (mean age ± SD, 67.3 ± 6.1; 52.2% women) were included in the analysis. Mean follow-up time was 8.7 years, during which 562 deaths occurred. Smoker and drinker accounted for 31.4% and 30.9%. The average WC and BMI were 82.5 ± 10.7 cm and 23.1 ± 3.7 kg/m^2^. More detailed baseline characteristic of the study participants was presented in Supplementary Table 1.

### Long‑term WC change trajectory

Results of group-based trajectory modeling analysis indicated that the model with four trajectories had a better fit (lowest Bayesian Information Criteria) than that with other number of trajectories. The four potential trajectories were identified and characterized as WC loss (a total average decrease of 8-12 cm), stable (change within 1 cm), moderate gain (increase of nearly 10 cm) and substantial gain (increase of nearly 20 cm).The group membership probability across 4 groups were  13.5%, 46.8%, 31.2%, and 8.5%. The average posterior probability assignment ranged from 78.6 to 86.2%, and indicating a good discrimination of trajectory. (Supplementary text, Supplementary Table 2 and 3).

The extracted four WC trajectories could be interpretable in context of the conventional WC categories (Fig. 1). Starting from the flattest to the steepest trajectory classes, WC stable trajectory was characterized with a slight WC change and covered 46.8% of participants. In the moderate gain trajectory, WC value increase by 10 cm and approximately entered into central obesity range. Another WC increase group (substantial gain) started out on a trajectory towards the greater end of obesity range. This trajectory covered 8.5% (*n* = 222) of participants with a rapidly total average gain of nearly 20 cm. The only decrease group (“loss”, *n* = 351) had an average decrease of 8–12 cm during follow-up period.

### Participant characteristics across WC change trajectory

The distribution of demographics and lifestyle characteristics across WC change trajectories was showed in Table 1. Participants with WC substantial gain trajectories were more likely to be women, rural residents and less educated, compared with stable groups. All physical measurement including WC/BMI, weight significantly disparate across the four trajectory groups. Higher proportion individuals with general/central obesity at baseline tended to follow WC loss pattern (*p* < 0.001). Relatively younger participants had higher odds of WC stable relative to other change group. However, their physical activity level, smoking and drinking status, dietary energy intake and duration of follow-up were similar. Additionally, participants in WC loss group tended to have a higher prevalence of chronic disease.

**Table 1:** Baseline characteristics of participants across WC change trajectory groups

*Demographics*	WC trajectories group				Statistics	*P*
	**Loss**	**Stable**	**Moderate gain**	**Substantial gain**		
***No. Participants***	351	1216	812	222		
***Follow-up duration (year)***	8.1 ± 5.0	8.6 ± 5.6	9.1 ± 5.7	8.8 ± 5.1	3.370	0.018
***No. Death during the follow-up,*** N (%)	80 (22.8)	232 (19.1)	184 (22.7)	66 (29.7)	14.058	0.003
Age (y) at baseline	68.0 ± 6.4	66.9 ± 5.8	67.1 ± 6.1	68.9 ± 7.0	8.420	< 0.001
Gender, N (%)						
Male	159 (45.3)	602 (49.5)	395 (48.7)	88 (39.6)	8.455	0.038
Female	192 (54.7)	614 (50.5)	417 (51.0)	134 (60.4)		
Location, N (%)						
Urban	200 (57.0)	612 (50.3)	361 (44.5)	94 (42.3)	20.363	< 0.001
Rural	151 (43.0)	604 (49.7)	451 (55.5)	128 (57.7)		
Education year (y) at baseline, N (%)	4.6 ± 4.9	5.1 ± 5.0	4.2 ± 4.6	3.9 ± 4.6	7.400	< 0.001
never	129 (40.3)	387 (34.5)	309 (41.3)	96 (47.1)	28.647	0.004
< 6 years	67 (20.9)	235 (21.0)	171 (22.8)	36 (17.7)		
6–8 years	32 (10.0)	158 (14.1)	85 (11.4)	27 (13.2)		
9–11 years	51 (15.9)	168 (15.0)	104 (13.9)	26 (12.8)		
> 11 years	41 (12.8)	173 (15.4)	80 (10.7)	19 (9.3)		
Income (yuan) at baseline	13,071.5 ± 16,000.6	11,786.0 ± 11,944.0	9936.5 ± 14,012.2	11,291.4 ± 17,779.3	3.780	0.010
***Behavior at baseline***						
Cigarette Smoker, N (%)	95 (27.2)	381 (31.4)	276 (34.0)	67 (30.3)	6.637	0.356
Alcohol Drinker, N (%)	99 (28.5)	385 (31.8)	263 (32.5)	56 (25.2)	5.715	0.126
Physical Activities (MET-hours/day)	7.8 ± 9.3	8.1 ± 10.3	7.2 ± 10.1	6.9 ± 9.6	1.960	0.119
***Anthropometry at baseline***						
Systolic blood pressure (mmHg)	136.9 ± 20.7	132.7 ± 20.1	130.2 ± 20.8	131.1 ± 21.7	8.980	< 0.0001
Diastolic blood pressure (mmHg)	84.0 ± 12.7	81.4 ± 12.2	80.0 ± 12.6	81.2 ± 12.6	8.530	< 0.0001
Height (cm)	158.8 ± 9.1	158.7 ± 8.6	157.7 ± 8.5	157.0 ± 8.6	3.920	0.008
Weight (Kg)	62.4 ± 11.9	60.1 ± 11.4	54.8 ± 10.0	52.8 ± 9.8	72.110	< 0.001
WC (cm)	92.5 ± 8.4	85.6 ± 9.0	76.4 ± 7.9	71.1 ± 7.2	494.600	< 0.001
Lean, N (%)	9(2.4)	203(16.7)	391(48.2)	147(66.1)	884.583	< 0.001
Normal, N (%)	46(13.0)	274(22.5)	246(30.3)	58(26.4)		
Morderate-high, N (%)	73(20.9)	378(31.1)	137(16.8)	17(7.5)		
High, N (%)	223(63.6)	361(29.7)	38(4.7)	0(0.0)		
BMI (kg/m^2^)	24.7 ± 4.2	23.8 ± 3.6	21.9 ± 3.1	21.4 ± 3.0	72.110	< 0.001
Lean, N (%)	27 (7.7)	76 (6.3)	103 (12.7)	45 (20.3)	208.553	< 0.001
Normal, N (%)	138 (39.3)	581 (47.8)	505 (62.2)	134 (60.4)		
Overweight, N (%)	124 (35.3)	411 (33.8)	181 (22.3)	38 (17.1)		
Obesity, N (%)	62 (17.7)	148 (12.2)	23 (2.8)	5 (2.3)		
***Dietary Total Energy (kcal) at baseline***	1956.0 ± 590.6	1988.5 ± 672.5	2038.6 ± 693.9	2005.1 ± 695.1	1.500	0.212
***Chronic disease***						
Diabetes	52 (14.8)	128 (10.5)	53 (6.5)	11 (5.0)	26.963	< 0.001
Myocardial infarction	26 (7.4)	54 (4.4)	35 (4.3)	7 (3.1)	7.401	0.060
Stroke	39 (11.1)	92 (7.6)	58 (7.1)	13 (5.9)	7.122	0.068
Cancer	2 (0.57)	22 (1.81)	10 (1.23)	1 (0.45)	4.979	0.173

Data shown as mean ± SD or N (%). Data was derived from China Health and Nutrition Survey (CHNS) from 1993 to 2015. Chi-square test for categorical variables and ANOVA for continuous variables were used to compare baseline characteristic differences. *WC* Waist circumference, *BMI* Body fat mass

### Association of WC change trajectories with mortality

**Fig. 2:**
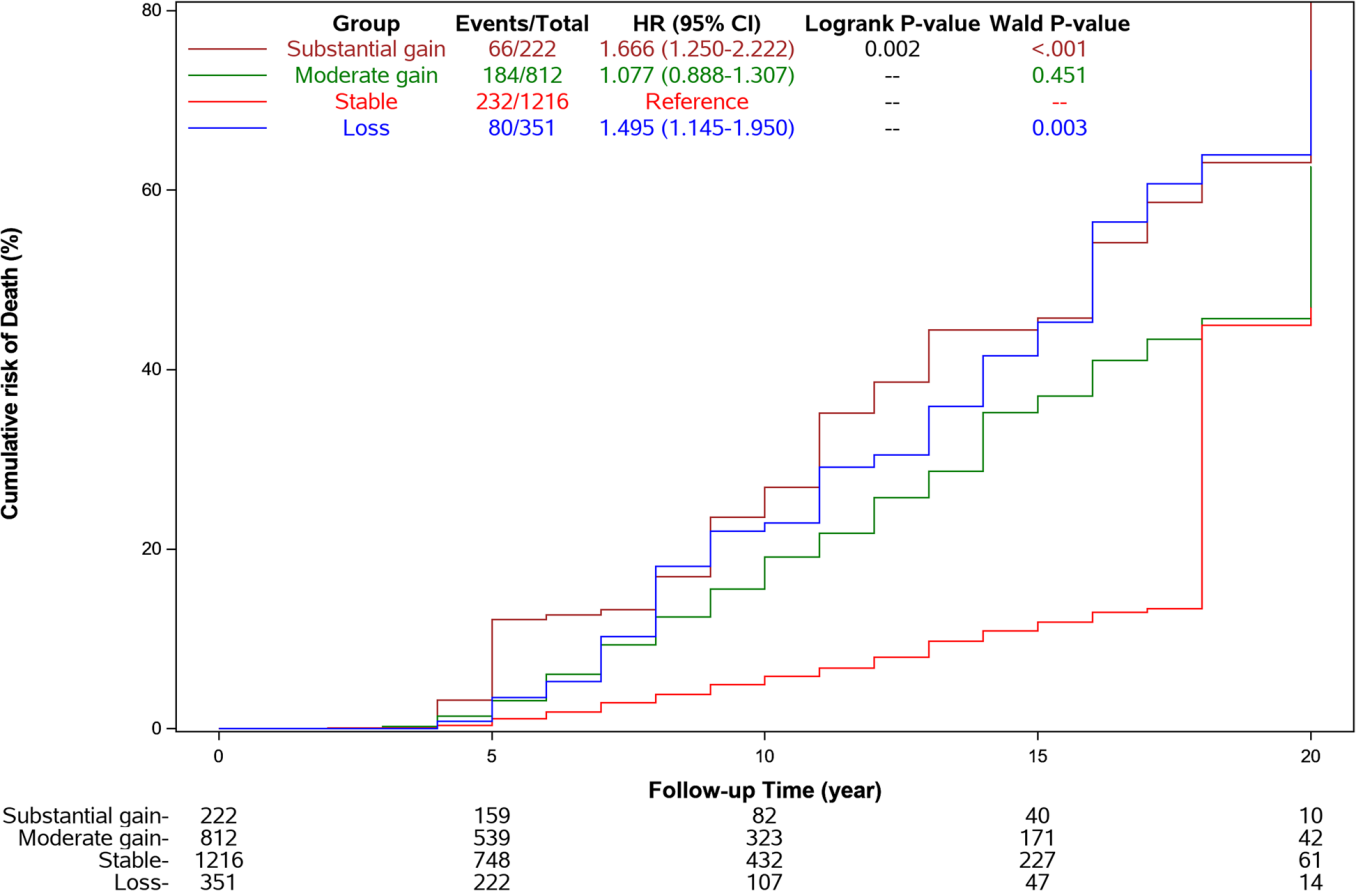
Cumulative incidence event risk of mortality among different patterns of waist circumference (WC) change trajectory

During the mean of 8.7 years of follow-up, a total of 562 death (21.6%) were reported. Among these participants, 80 were in WC loss group (22.8%), 232 in “stable” group (19.1%), 184 in “moderate gain group” (22.7%) and 66 in “substantial gain” group (29.7%). Cumulative hazard of mortality across the four trajectory patterns is shown in Fig. 2. Both loss and substantial gain patterns were associated with increased risk of mortality (Table 2). Compared with the stable group, substantial WC gain and loss group witnessed 55% (HR: 1.55, 95%CI: 1.18–2.04) and 38% (HR: 1.38, 1.07–1.79) increase in the risk of mortality. The association was somewhat attenuated but persisted significance after adjustment for age, gender, PA level and other potential covariates. No significant relation were observed between moderate gain group and mortality (HR: 1.07, 95%CI: 0.88–1.30; *p* = 0.512).

**Table 2:** Multivariate Cox regression analysis for the associations between WC change trajectories and mortality ^a^

Models	Loss		Moderate gain		Substantial gain	
	HR(95%CI)	*P*	HR(95%CI)	*P*	HR(95%CI)	*P*
Model 1	1.38 (1.07–1.79)	0.014	1.07(0.88–1.30)	0.512	1.55(1.18–2.04)	0.002
Model 2	1.34(1.01–1.78)	0.041	1.13(0.91–1.41)	0.253	1.54 (1.12–2.12)	0.008
Model 3	1.45 (1.11–1.90)	0.007	1.07 (0.88–1.30)	0.505	1.62(1.22–2.16)	0.001

^a^HR, hazard ration. CI, confidence interval, WC stable trajectory was reference group

Model 1 was unadjusted model without any covariates

Model 2 adjusted age, gender, enrollment year, education and income level, smoking and drinking status, physical activity levels, initial BMI and WC, initial SBP/DBP, dietary energy intake and chronic disease

Model 3 adjusted propensity score of aforementioned covariates as a linear and only covariate

### Joint analysis of WC change trajectories and baseline BMI

We further assessed the joint association of WC change trajectories and initial BMI with mortality (Table 3). Individuals in normal BMI at baseline and maintained stable WC were assigned as reference. Compared to the reference group, adults with normal weight but following WC substantial gain pattern were at 54% increased risk of mortality after controlling for potential confounders (HR:1.54, 95%CI: 1.10–2.17). While the adverse effect of WC loss weaken to insignificance among normal weight group (*p* = 0.113). The risk of mortality generally increased for all WC change group among baseline overweight individuals, and those in WC loss group were at higher risk (HR:2.43, 95%CI: 1.41–4.19; *p* = 0.001). The HRs for WC stable pattern, moderate gain and substantial gain were 1.67 (1.07–2.60), 1.58 (0.95–2.63) and 1.36 (0.60–3.09) respectively.

**Table 3:** The joint analysis of initial BMI and WC change trajectories on risk of mortality

Group	Crude model		Adjusted model	
	HR(95%CI)	*P*	HR(95%CI)	*P*
Non-overweight				
Loss	1.67(1.20–2.33)	0.002	1.35 (0.93–1.94)	0.113
Stable	Ref		Ref	
Moderate gain	1.09(0.87–1.36)	0.446	1.06 (0.83–1.35)	0.636
Substantial gain	1.63(1.20–2.21)	0.002	1.54 (1.10–2.17)	0.013
Overweight and obesity				
Loss	1.19(0.81–1.73)	0.378	2.43 (1.41–4.19)	0.001
Stable	0.93(0.71–1.21)	0.580	1.67 (1.07–2.60)	0.025
Moderate gain	0.87(0.61–1.25)	0.460	1.58(0.95–2.63)	0.080
Substantial gain	0.97(0.80–1.16)	0.704	1.36(0.60–3.09)	0.458

Data shown as HR (95%CI). Non-overweight was defined as BMI at baseline < 24.0 kg/m^2.^, Overweight was defined as BMI at baseline > 24.0 kg/m^2^. *HR* Hazard ration. *CI* Confidence interval. WC stable trajectory and initial non-overweight was the reference group

Crude model adjusted no covariate; adjusted model included age, gender, enrollment year, education and income level, smoking and drinking status, physical activity levels, initial SBP/DBP, dietary energy intake and chronic diseases

### Subgroup analysis and sensitivity analysis

No significant interaction was observed between prespecified subgroup and mortality (p for interaction > 0.05; Supplemental Fig. 2). These different strata all provided consistent results with our main findings. Sensitivity analysis by excluding the participants with measurement < 4 times suggested no disparate results with those from the analytic samples. Similar finding was observed in multiple imputation datasets, with the direction and magnitude of the association persisted. However, when we excluded individuals with history of chronic disease at baseline, the adverse effect of WC loss trajectory attenuated to no statistical significance (HR: 1.43, 0.96–2.14; *p* = 0.082). Detailed results was presented in Supplementary Table 4. Significant higher risk of mortality was observed in non-central obesity participants following WC substantial trajectory (HR: 1.49, 95%CI: 1.09–2.04) and those in high WC level and WC loss pattern (HR: 1.59, 95%CI: 1.12–2.26), compared with individuals in normal WC level and persisted stable trajectory (Supplementary Table 5).

## Discussion

In this prospective cohort study of older Chinese, we identified 4 distinct WC change trajectories by latent trajectory method. The study found that WC change was associated with an increased all-cause mortality, with a  54% increased for long-term WC substantial gain and  34% increased for WC loss, compared with stable WC. By comparison, the effect of moderate gain was more modest and insignificant (7% increase in mortality). Furthermore, the increased risk was more striking for those in WC loss and overweight group at baseline, compared with WC stable and normal BMI. Our study suggested avoiding excessive WC gain or loss and maintaining reasonable weight might be helpful to reduce risk of mortality. However, the results should be interpreted with caution due to the small sample size and complexity of WC change in the older. Further study was warranted to confirm our findings and clear the possible reasons.

We extracted four distinct and mutually exclusive WC change pattern using LCTA method and assessed the possible contributors of baseline characteristic or behaviors to their trajectory profiles. This helps to conceptualize the change trajectory of WC and probe into the individuals’ heterogeneity in the susceptibility of change over time, which is helpful for public health policy and further research. During the 8.7-year period, over a third of participants gained WC significantly and transited up into higher level over time. It might be likely that these numbers would be higher for today’s adults, as abdominal obesity is highly prevalent among Chinese adults, compounded by the ageing of the population [15, 32]. Our analysis suggested that women were more likely to follow WC substantial gain trajectory. Previous studies reported consistent finding that women showed a great increase in WC than men of the same age, and continued increase their WC throughout the lifespan [5, 33]. It has been hypothesized that hormonal levels might be responsible for post-menopausal changes in fat distribution [34]. In addition, relatively older or less educated participants had higher odds of WC loss or substantial gain, compared with stable group. Participants in WC loss trajectory were more likely to be overweight or obese at baseline. Both intentional and unintentional weight loss could occur in older population, and they might be associated with disparate health outcomes [35]. However, We could not evaluate the potential intention for the loss limited by data and further study was required to address this.

Our data suggested that the bulk of adverse changes in mortality among older Chinese might be attributable to excessive rapid WC gain, irrespective of main confounders including age, initial BMI and health-related behavior factors. The adverse effect persisted across subgroup and sensitivity analysis. This finding was in accordance with previous cohort studies in other countries [7, 23, 36]. Actually, in a meta-analysis of 26 cohort studies, weight gain were associated with 45% higher mortality risk in middle aged and older adults [37]. It was well known that WC strongly reflected visceral adipose tissue, a risk factor for certain cancers and cardiometabolic disturbances [38]. Inflammation of visceral adipose tissue mediated metabolic disturbances [39], and excess adipose tissue elevated free fatty acid release, which might result in cellular proliferation and tumor growth [40]. Another sobering finding was that participants who gain WC moderately and entered into central obesity range tended to be accompanied by no lower risk of mortality. This was incongruent with previous epidemiological data where “obesity paradox” phenomenon was observed [19, 41, 42]. According to this paradox, a survival benefit was reported among overweight individuals compared with those in healthy weight. It has been proposed that overweight/obesity might provide a metabolic buffer for wasting diseases in older subjects [27]. Another explanation for the discrepant results might be reverse causality or residual confounding, as the paradox were attenuated in studies with short follow-up duration or in those including smokers [43].

The positive association between WC loss trajectory and all-cause mortality was consistent with previous cohort studies in different cultural and socioeconomic population [9, 26]. Results from the Melbourne collaborative cohort study reported that WC loss was associated with a 26% (HR: 1.26, 95%CI: 1.09–1.47) increased risk of all-cause mortality, compared to those who had minimal changes [9]. WC loss in older population might be an indicator encompassing not just fat but also loss of skeletal muscle and bone [44], thus WC loss in later life might be an increasing frailty or underlying disease resulting in excess mortality [37]. In China, WC loss related morbidities including cancer, diabetes, and kidney disease were the cause of death [45]. In addition, undernutrition, low-grade inflammation and sarcopenia might also explained partly the enhanced risk of mortality [46]. However, there was increased risk of reverse causation in the aged population due to the high prevalence of most chronic diseases. In our sensitivity analysis by excluding subjects with WC measurement < 4 times, the positive WC-mortality relations weakened to the marginal statistical significance and disappeared if excluding those with chronic disease. More work was needed to establish the actual association and explore the possible mechanism.

Of note, the current study suggested that the effect of WC change stratified by initial BMI, and the striking increased risk was observed for WC loss and stable in overweight participants, providing evidence that baseline BMI might be an effect modifier. Similar joint association between BMI and WC was previously reported [11, 23]. The adverse effect of WC substantial gain persisted in normal BMI but decreased to in insignificance in overweight population, compared with normal weight and stable WC group. Those with normal BMI and high WC might have excessive visceral fat but less muscle mass, compared with individuals in same BMI weight and no central obesity [47]. Nevertheless, the no association in those with higher baseline BMI should be interpreted in caution, as the robustness of results might be limited by the small sample (*N* = 43). Additionally, initially overweight and stable WC also contributed to higher risk of mortality. Similar moderating effect was also observed between baseline WC and WC change patterns in sensitivity analysis. Taken together, the joint analysis indicated that individuals with normal weight and maintaining stable or moderate WC change had lower mortality risk than other groups. Given the small sample of each group, further examination of whether and how baseline BMI/WC modifies the association between WC change and mortality in older adults was essential.

Several strengths and limitations should be addressed when interpreting the our findings. The longitude analysis of WC change patterns and their interaction with BMI at baseline might be of great significance to promote prevention adiposity issue among Chinese older adults. The LCTA trajectory method had the unique ability to extract the developmental course of WC and categorize individuals into distinct, mutually exclusive groups. All physical measurements were conducted by research staff, which reduced cognitive biases because of self-administered measurements. However, the sample size might limit the results robustness for estimating mortality risk, although the main results were consistent with previous studies across Asian and western countries [23, 36]. Second, the association between WC change trajectories and specific-cause mortality was not evaluated owing to lack of data. Third, residual confounding cannot be fully avoided, although we adjusted for detailed covariates including chronic diseases. But the adverse effect of WC loss and substantial gain persisted across a series of sensitivity, which might argue against reverse causality to some extent. Forth, we could not specify the reasons for weight change or differentiate whether they were intentional or not. It generally considered that weight loss regardless of intentional or unintentional was associated with adverse diseases and characteristics in older men, and suggested that the potential intentions such as personal choice, intentionally physician’s advice, intentionally or unintentionally owing to ill health might be associated with disparate morality risk [35, 48]. We could answer these knowledge in current study limited by data and further work was warranted to assess our findings and identify the possible biological pathways between long-term WC change and mortality.

## Conclusions

Our study suggested that majority of participants suffered WC change during follow-up in older Chinese. Both long-term WC loss and substantial gain was associated with increased risk of mortality, with some evidence that overweight individuals might have a greater risk imposed by WC loss than those in normal BMI, although the mechanism was not well understood. Taken together, our study suggested avoiding excessive weight gain and maintaining stable WC might be beneficial for mortality. Due to that there were relatively few end events in each subgroup, the interpretation of results should be cautious. Further studies were required to unravel the mechanism underlying the association between WC change and mortality and to confirm our findings.

## Supplementary Information

**Additional file1.**

## Data Availability

The datasets analyzed in the current study are available from the corresponding author on reasonable request.

## Abbreviations

BMI: Body mass index
BIC: Bayesian Information Criterion
CHNS: China Health and Nutrition Survey
CIs: Confidence internal
HRs: Hazard ratios
LCTA: Latent class trajectory analysis
WC: Waist circumstance
