# Supplementary material for: Long-term waist circumference trajectories and body mass index with all-cause mortality in older Chinese adults: a prospective nationwide cohort study
Source: Arch Public Health. 2022 Sep 10;80:94. doi: 10.1186/s13690-022-00861-y (PMC9463814; doi:10.1186/s13690-022-00861-y)

**Supplementary ·Text. Fitting** **information on the fitting of WC trajectory model and parameter estimation**

Using a maximum-likelihood approach, LCTA estimated multiple regression models simultaneously and calculated from the model parameters the probability of each participants belonging to each trajectory group. The model fitting procedure of WC change trajectory with Proc Traj is listed as follows:

**Step 1**. Decide on the optimal number of groups using substantive knowledge. Based on previous researches (1, 2) and our substantive knowledge about the long-term change of WC characteristics, we decided to fit two- to five-group models of WC change trajectories.

**Step 2**. Fit number of groups to data. To determine the optimal number of trajectory groups included in the model, we compared Bayesian Information Criteria (BIC) between models with different groups. A smaller BIC value indicated a better fit(3), but as the number of groups was increased across models, the additional reduction of BIC became smaller (**Supplementary Table S1**). Following the existing guidelines(4, 5), we chose the model when additional groups failed to increase BIC by at least half of the BIC in the previous model and the value of group membership probability ≥5%.

**Step 3**. Select the shape of the pattern of change for each group over time. After the number of groups was selected, we determined further the shape of each trajectory group using a stepwise approach in establishing polynomial order, with all groups initially set to cubic order. As the order of each trajectory was set to quadratic, linear, and intercept respectively, we compared the changes in BIC and the significance of parameters across models. Based on the substantive knowledge and statistical inference, the cubic trajectory pattern with four trajectory groups was regarded as the best fitting for the data.

Once a model was selected, we examined the posterior probabilities for each trajectory group to ensure all groups provided evidence for adequate model fitting. A general rule required an acceptable model to have a minimum average posterior probability of 0.70 for all trajectories(4). In this study, average posterior probabilities for all trajectories were at least 0.75 (**Supplementary Table S2**)

**References**

1. Ye M, Robson PJ, Eurich DT, Vena JE, Xu JY, Johnson JA. Changes in body mass index and incidence of diabetes: A longitudinal study of Alberta's Tomorrow Project Cohort. Preventive medicine. 2018;106:157-63.

2. Wang M, Yi Y, Roebothan B, Colbourne J, Maddalena V, Wang PP, et al. Body Mass Index Trajectories among Middle-Aged and Elderly Canadians and Associated Health Outcomes. J Environ Public Health. 2016;2016:7014857.

3. Nagin DS. Analyzing developmental trajectories: a semiparametric, group-based approach. Psychological methods. 1999;4(2):139.

4. Nagin DS, NAGIN D. Group-based modeling of development: Harvard University Press; 2005.

5. Jones BL, Nagin DS, Roeder K. A SAS procedure based on mixture models for estimating developmental trajectories. Sociological methods & research. 2001;29(3):374-93.

**Supplementary Table 1. Baseline characteristic of participants from CHNS 1993-2015**

| ***Demographics*** | ***N*** | ***%*** |
| --- | --- | --- |
| ***No. Participants*** | 2601 |  |
| ***Follow-up duration (year)*** | 8.7±5.5 | |
| ***No. Death during the follow-up*** , N (%) | 562 | 21.6 |
| Age (y) at baseline | 67.3±6.1 | |
| Gender, N (%) |  |  |
| Male | 1244 | 47.8 |
| Female | 1357 | 52.2 |
| Urbanization, N (%) |  |  |
| Urban | 1285 | 48.7 |
| Rural | 1346 | 51.3 |
| Education year (y) at baseline, N (%)* | 4.7±4.8 | |
| never | 921 | 38.5 |
| <6 years | 509 | 21.3 |
| 6-8 years | 302 | 12.6 |
| 9-11 years | 349 | 14.6 |
| >12 years | 313 | 13.1 |
| Income (yuan) at baseline | 11557.6±14219.5 | |
| ***Behavior at baseline*** |  |  |
| Cigarette smoker, N (%)* | 819 | 31.4 |
| Alcohol drinker, N (%)* | 803 | 30.9 |
| Physical activities (MET-hours/day) | 7.8±10.1 | |
| ***Anthropometry at baseline*** |  |  |
| ***Systolic blood pressure (mmHg)*** | 132.5±20.6 | |
| ***Diastolic blood pressure (mmHg)*** | 81.4±12.5 | |
| ***Height (cm)*** | 158.3±8.7 | |
| ***Weight (Kg)*** | 58.3±11.5 | |
| ***WC (cm)*** | 82.5±10.7 | |
| ***BMI (kg/m***^2^) | 23.1±3.7 | |
| Lean, N (%) | 251 | 9.7 |
| Normal, N (%) | 1358 | 522 |
| Overweight, N (%) | 754 | 28.9 |
| Obesity, N (%) | 238 | 9.2 |
| ***Dietary Total Energy (kcal) at baseline*** | 1999.2±670.3 | |

Values are mean ± SD or N (Percent). *Some missing values for this category.

**Supplementary Table 2. Tabulated Bayesian Information Criterion (BIC) for all participants**

| **Number of Groups** | **BIC** | Group membership probability (%) | | | | |
| --- | --- | --- | --- | --- | --- | --- |
|  |  | **Group 1** | **Group 2** | **Group 3** | **Group 4** | **Group 5** |
| 2 | -209771.7 | 46.11 | 53.89 |  |  |  |
| 3 | -206139.7 | 19.84 | 53.89 | 26.27 |  |  |
| **4** | **-203980.6** | **9.2** | **37.51** | **40.79** | **12.49** |  |
| 5 | -2032887.7 | 3.82 | 19.15 | 37.09 | 30.71 | 9.22 |

**Supplementary Table 3. Parameters estimated for WC trajectory change pattern**

| WC trajectory change pattern | Parameter Estimate (SE)^a^ | | | | Group membership probability (%) | Average posterior probability (%) |
| --- | --- | --- | --- | --- | --- | --- |
|  | Intercept term | Linear term | Quadratic term | Cubic term |  |  |
| Loss | -0.389 (0.131) | -3.751 (0.096) | 2.358 (0.012) | -0.009 (0.001) | 9.2 | 86.2 |
| Stable | -0.152 (0.062) | -0.121 (0.023) | 0.014 (0.001) |  | 37.5 | 78.6 |
| Moderate gain | 0.144 (0.062) | 1.162 (0.038) | -0.053 (0.005) | 0.001 (0.000) | 40.8 | 77.8 |
| Substantial gain | 1.080 (0.108) | 2.243 (0.035) | -0.063 (0.002) |  | 12.5 | 83.9 |

^a^ Parameter estimate presented the shape of each pattern of trajectory over time. Intercept term interpreted as the expected level of change of WC

in cm at the first year of follow-up. Linear term interpreted as the linear slope of change of WC by follow-up year. Quadratic term interpreted as the quadratic slope and cubic term interpreted as the cubic slope.

**Supplementary Table4. Sensitivity analysis of the associations between different patterns of WC trajectories and the risk of mortality** ^a^

| Datasets | Loss | |  | Moderate gain | |  | Substantial gain | |
| --- | --- | --- | --- | --- | --- | --- | --- | --- |
|  | HR(95% CI) ^b^ | *P* value |  | HR(95% CI) ^b^ | *P* value |  | HR(95% CI) ^b^ | *P* value |
| Exclude WC measurement <4 times | 1.38(1.00-1.92) | 0.049 |  | 1.09 (0.87-1.36) | 0.465 |  | 1.89(1.35-2.64) | <0.001 |
| Exclude chronic disease | 1.43(0.96-2.14) | 0.082 |  | 1.21(0.94-1.54) | 0.135 |  | 1.92 (1.34-2.74) | <0.001 |
| Multiple Imputation | 1.36(1.03-1.79) | 0.029 |  | 1.06(0.86-1.31) | 0.576 |  | 1.52(1.12-2.07) | 0.007 |

^a^ all models adjusted variables including adjusted age, gender, enrollment year, urbanization, education and income level, smoking and drinking status, physical activity levels, initial BMI and WC, initial SBP/DBP, dietary energy intake.

^b^ HR, hazard ration. CI, confidence interval

**Supplementary Table 5. The joint analysis of initial WC and WC change trajectories on risk of mortality**

| Group | N | Crude model | | Adjusted model | |
| --- | --- | --- | --- | --- | --- |
|  |  | HR(95%CI) | *P* | HR(95%CI) | *P* |
| Non-central obesity |  |  |  |  |  |
| Loss | 68 | 1.45(0.91-2.29) | 0.116 | 1.34(0.84-2.14) | 0.224 |
| Stable | 537 | Ref. |  | Ref. |  |
| Moderate gain | 677 | 1.09(0.87-1.38) | 0.441 | 1.09(0.86-1.37) | 0.479 |
| Substantial gain | 209 | 1.47(1.09-1.99) | 0.012 | 1.49(1.09-2.04) | 0.011 |
| Central obesity |  |  |  |  |  |
| Loss | 283 | 1.36(1.00-1.86) | 0.054 | 1.59(1.12-2.26) | 0.010 |
| Stable | 679 | 0.86(0.65-1.14) | 0.289 | 1.06(0.77-1.46) | 0.725 |
| Moderate/ substantial gain* | 148 | 0.62(0.37-1.03) | 0.612 | 0.84(0.48-1.46) | 0.526 |

Non-central obesity was defined as WC at baseline<88.0 cm in male or 80.0 cm in females, central obesity was defined as WC at baseline ≥ 88.0cm in males or ≥ 80.0 cm in females.

HR, hazard ration. CI, confidence interval. WC stable trajectory and initial non-central obesity was the reference group.

Crude model adjusted no covariate; adjusted model included age, gender, enrollment year, education and income level, smoking and drinking status, physical activity levels, initial SBP/DBP, dietary energy intake and chronic diseases.* The substantial gain was merged with moderate gain group, because of the small sample size (N of substantial group was 13).

**Supplementary Figure 1. Flow diagram for cohort selection and censure**


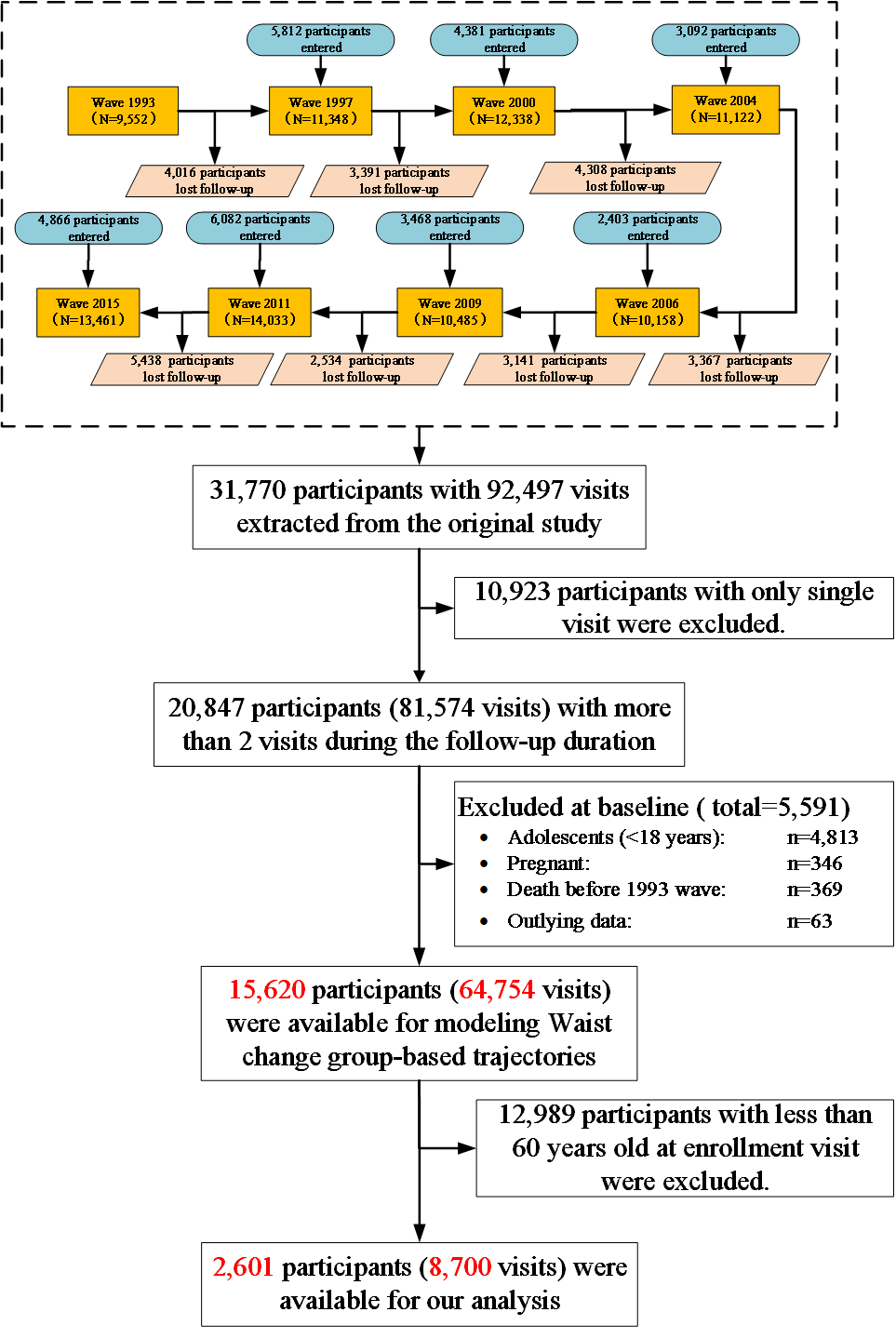


**Supplementary Figure 2. Subgroup analysis for the associations between different WC change trajectories and risk of mortality**


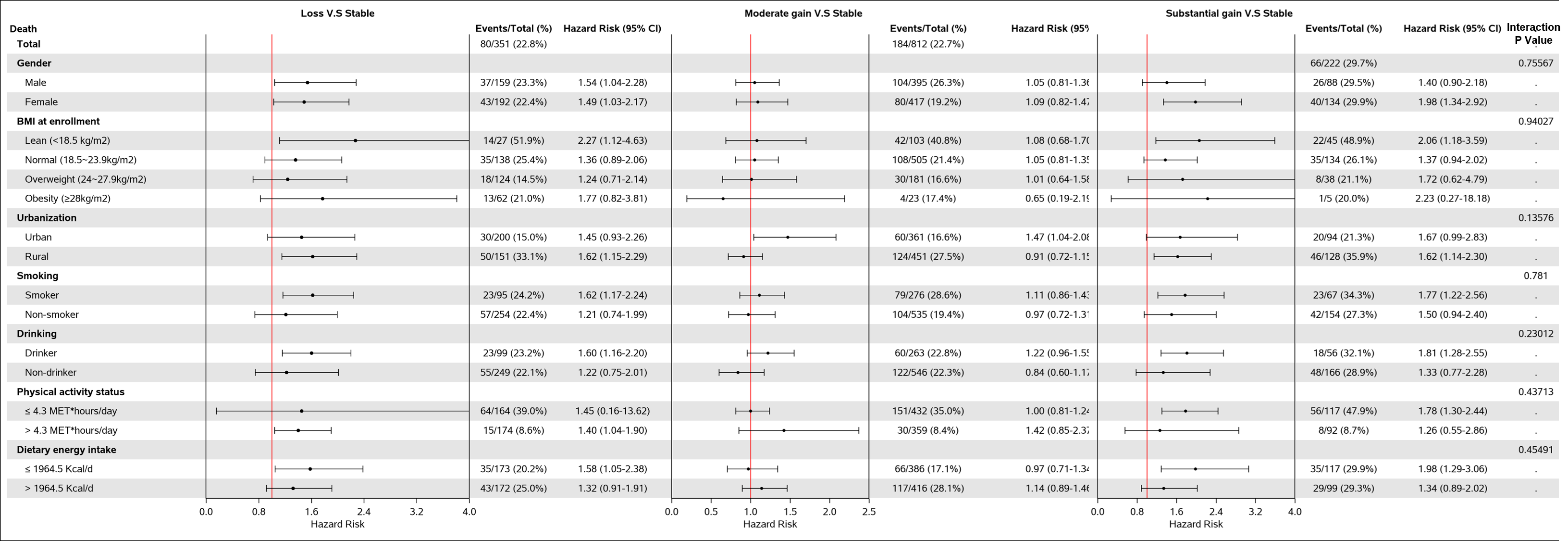

Supplement: Supplementary file 1 — Additional file1. [file 13690_2022_861_MOESM1_ESM.docx]
